# Editorial: Multimodal perceiving technologies in neuroscience and vision applications

**DOI:** 10.3389/fnins.2024.1477572

**Published:** 2024-08-28

**Authors:** Avid Roman-Gonzalez

**Affiliations:** ^1^Aerospace Sciences & Health Research Laboratory, Universidad Nacional Tecnológica de Lima Sur, Lima, Peru; ^2^Universidad Nacional de Moquegua, Moquegua, Peru

**Keywords:** brain computer interface (BCI), neuroscience, human machine integration, human machine interface (HMI), electroencephalography

Advancements in neuroscience and vision applications rapidly transform our understanding of the brain and its intricate functions. The Research Topic “*Multimodal perceiving technologies in neuroscience and vision applications*” brings together pioneering research that leverages multimodal approaches to deepen our insight into neural mechanisms and vision-related disorders. This editorial aims to frame the objectives of this Research Topic and contextualize its significant findings.

Multimodal perceiving technologies integrate various data sources, such as EEG, MRI, and computational models, to comprehensively understand neural processes. These technologies have proven invaluable in capturing the complexity of brain functions and disorders. The contributing articles in this Research Topic exemplify the innovative application of multimodal methods in neuroscience and vision research.

The article “*Brain-inspired modular echo state network for EEG-based emotion recognition*” delves into developing a novel computational model inspired by brain modularity (Yang et al.). This research presents a modular echo state network that effectively recognizes emotions from EEG signals, showcasing the potential of bio-inspired models in emotion detection. By leveraging the intricate architecture of the brain, this study opens new pathways for enhancing the accuracy and efficiency of emotion recognition systems.

In “*3.0 T multi-parametric MRI reveals metabolic and microstructural abnormalities in the posterior visual pathways in patients with thyroid eye disease*”, the authors utilize advanced MRI techniques to uncover metabolic and structural alterations in patients with thyroid eye disease (Luo et al.). This comprehensive MRI approach highlights the importance of multi-parametric imaging in diagnosing and understanding visual pathway abnormalities. The findings underscore the potential of high-field MRI in clinical diagnostics and its role in advancing personalized medicine.

The article “*A protocol to quantify cross-sectional and longitudinal differences in duction patterns*” introduces a robust protocol for assessing eye movement patterns (Willeford et al.). This research is crucial for understanding various vision disorders and their progression. By providing a standardized method to quantify duction patterns, the study offers a valuable tool for clinicians and researchers to monitor and evaluate the efficacy of treatments over time.

Finally, “*Attention-based 3D convolutional recurrent neural network model for multimodal emotion recognition*” presents an innovative neural network model that integrates attention mechanisms with 3D convolutional and recurrent layers (Du et al.). This multimodal approach significantly improves emotion recognition by combining spatial and temporal features from various data sources. The study demonstrates the effectiveness of attention-based models in handling complex, multimodal data for emotion detection.

These articles illustrate the transformative impact of multimodal perceiving technologies on neuroscience and vision applications. They highlight the integration of advanced computational models, sophisticated imaging techniques, and standardized protocols in uncovering the brain's and visual system's complexities.

The broader implications of this research are profound. By improving our understanding of neural and visual disorders, these studies pave the way for more effective diagnostic tools, personalized treatments, and innovative therapeutic approaches. Moreover, integrating multimodal technologies fosters interdisciplinary collaboration, driving progress in neuroscience and vision research.

As we continue to explore the potential of multimodal perceiving technologies, we must recognize the challenges and opportunities they present. Future research should focus on refining these technologies, enhancing their accessibility, and ensuring their ethical application in clinical and research settings. By addressing these challenges, we can unlock the full potential of multimodal approaches and usher in a new era of discovery in neuroscience and vision science.

In conclusion, the Research Topic “*Multimodal perceiving technologies in neuroscience and vision applications*” showcases cutting-edge research that advances our understanding of the brain and vision. The contributing articles exemplify the innovative application of multimodal methods, offering valuable insights and paving the way for future advancements in the field. We hope this Research Topic of studies inspires further research and collaboration, ultimately contributing to improving human health and wellbeing.

